# Dense Crowd Dynamics and Pedestrian Trajectories: A Multiscale Field Dataset from the Festival of Lights in Lyon

**DOI:** 10.1038/s41597-025-04732-3

**Published:** 2025-04-30

**Authors:** Oscar Dufour, Huu-Tu Dang, Jakob Cordes, Raphael Korbmacher, Mohcine Chraibi, Benoit Gaudou, Alexandre Nicolas, Antoine Tordeux

**Affiliations:** 1 Université Claude Bernard Lyon 1, CNRS, Institut Lumière Matière, UMR5306, F-69100 Villeurbanne, France; 2 UMR 5505 IRIT, Université Toulouse Capitole, Toulouse, France; 3 Institute of Advanced Simulation, Forschungszentrum Jülich GmbH, Jülich, Germany; 4 Institut für Theoretische Physik, Universität zu Köln, Köln, Germany; 5 Fakultät für Maschinenbau und Sicherheitstechnik, Bergische Universität Wuppertal, Wuppertal, Germany

**Keywords:** Applied physics, Systems biology

## Abstract

The dynamics of dense crowds have received considerable attention from researchers seeking fundamental understanding or aiming to develop data-driven algorithms to predict pedestrian trajectories. However, current research mainly relies on data collected in controlled settings. We present one of the first comprehensive field datasets describing dense pedestrian dynamics at different scales, from contextualized macroscopic crowd flows over hundreds of meters to microscopic trajectories (around 7000 individual trajectories). In addition, a sample of GPS traces, some statistics of contacts and pushes, and a list of non-standard crowd phenomena observed in the video recordings are provided. The data were collected during the 2022 Festival of Lights in Lyon, France, as part of the French-German MADRAS project and cover densities up to 4 pedestrians per square meter. We suggest using this extensive dataset, acquired in complex real-world settings, to benchmark models of pedestrian dynamics.

## Background & Summary

Large gatherings raise challenges regarding public safety and flow management; these challenges tend to be all the more acute as the crowd is large and dense. Religious festivals, music concerts, and significant outdoor events are therefore of particular concern, with a record of poor crowd management and, in the worst cases, deadly crowd crushes^1–4^. Beyond the rules of thumb that have been refined over the years, a deeper fundamental understanding of the dynamics of dense crowds will be instrumental for more efficient event planning and crowd management. Here, a crowd will be described as dense if its density exceeds the arbitrary threshold of 1.5 or 2 ped/m^2^, thus falling in Fruin’s Level of Service^5^; critical conditions with extreme densities (above 8 ped/m^2^), which must be avoided in practice, are left out of our scope.

The present theoretical grasp of dense crowd dynamics mainly stems from controlled experiments^6^, conducted in idealized settings. This protocol enables researchers to finely control variables and settings to observe a whole gamut of none-too-common scenarios, such as emergency evacuations^7^ or high-density flows and crossings. The Research Center of Jülich, in particular, has collected numerous pedestrian trajectories in a wide array of such experiments^8,9^. Let us also mention, among others, the datasets collected by the groups of Haghani^10^ and Zuriguel^11^ to explore crowd dynamics during emergency evacuation drills, Murakami *et al*.^12^ to probe the emergence of unidirectional and bidirectional pedestrian flows. An open-access data archive can be found here^13^. However, the controlled conditions may substantially differ from reality, and the participants are aware of their involvement in the research. Another drawback of controlled experiments is that they do not afford a comprehensive picture of the crowd; the broader context in which the dynamics occur is missing.

The thirst for *field data* to train data-based methods, such as machine learning algorithms, remains unquenched for dense crowds: field studies typically involve situations of low density. For instance, the widely used ETH^14^ and UCY^15^ datasets, which originate from surveillance videos, capture pedestrian scenes at density 0.1-0.5 ped/m^2^, with many avoidance situations. In this regime, the pedestrian dynamics are believed to be governed by different mechanics than at higher density^5,16,17^. Empirical datasets often encompass a heterogeneous mix of road users, as in the Stanford Drone Dataset (SDD)^18^, including pedestrians, cyclists, skateboarders, cars, and buses. While these datasets capture small scenes, the Grand Central Station Dataset (GS)^19^, collected in New York, covers a vast area. Although one scene can contain hundreds of pedestrians, the average density is below 0.2ped/m^2^ due to the region’s size. The Edinburgh Informatics Forum Dataset (EIF)^20^ provides trajectories of 92 000 people in a university playground from an overhead camera, with minute average densities (most of the time just a few pedestrians at a time). Also worth mentioning are mass-gathering studies relying on a sparse sample of smartphone signals^21^. Besides, the group of Toschi and Corbetta at the Technical University of Eindhoven have collected extensive datasets of pedestrian trajectories in the Eindhoven train station and on the university campus in the last decade^22,23^. Several reviews on existing field studies of pedestrian trajectories have recently appeared^24–26^.

The present contribution aims to make up for the lack of field data on dense crowds by providing a comprehensive picture of pedestrian flows at a large gathering around a hot spot of a major cultural and entertainment event, the 2022 edition of the Festival of Lights, which took place in Lyon, France. For this purpose, we collected various data relevant to pedestrian dynamics and crowd management. These data cover an extensive range of length scales, by all standards in the field, from the global flow picture and contextual elements down to individual pedestrian trajectories and some statistics on physical contacts. In the following, an emphasis shall be placed on all observations that depart from what is typically prescribed or observed in controlled experiments, thus further highlighting the added value of actual field data.

**Fig. 1:**
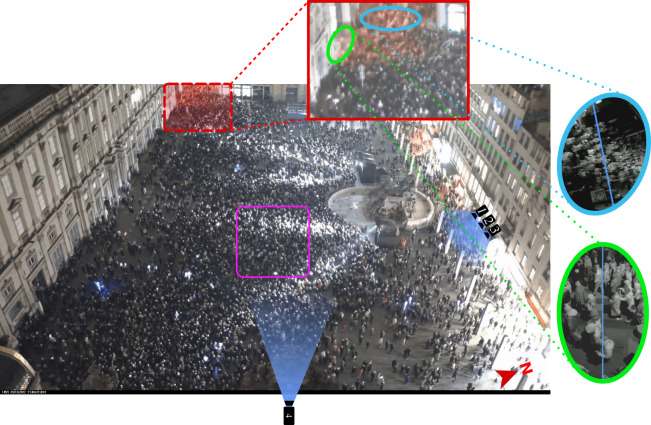
The thick crowd gathered in and around *Place des Terreaux* at the end of a light show on December 12, 2022, at 9:41 PM. The cameras’ fields of view are highlighted in blue; the pink rounded square delimits the area where pedestrians were exhaustively tracked in the *LargeView* datasets^30^, *LargeView* Trajectories^37^. Inset: Zoom on two exit streets where we monitored the outflow.

Lyon’s yearly Festival of Lights is a four-evening event (from December 7 to 11 in 2022, mostly from 7 pm to 11 pm) wherein the city is lit up remarkably. Originally a religious tribute to the Virgin Mary, it has become a massive international festival renowned for its innovative light shows and artistic projections on historic buildings. The event attracts millions of local and international visitors (more than 2 million officially in 2022^27^). Key attractions include *Place des Terreaux* and *Place Saint-Jean*, reportedly attended by 150 000 and 80 000 spectators every night, respectively, in 2022^27^. Quite interestingly, for our purposes, managing the associated crowd flows is one of the most prickly issues for the event organizers^28^. They aim to ensure smooth flows and reasonable delays for a pleasant experience. Still, above all, to ward off crowd accidents, after a difficult situation witnessed in the 2000s (information obtained from a private communication with the organizers of the event) and the tragedies that have occurred in massive entertainment events around the world, e.g. at the Love Parade in Duisburg, Germany, in 2010^2^ or during Halloween on the streets Seoul, Korea, in 2022^29^. This is achieved by regulating flows at different scales: macroscopically, by suggesting routes through the city to visit the multiple light installations, for instance, starting at *Place Bellecour*, moving to *Place des Terreaux*, and then exploring *Vieux Lyon*, especially near *Saint-Jean’s Cathedral*; mesoscopically, by installing barriers and safety agents, particularly near *Place des Terreaux* and imposing unidirectional flows in many streets to ease congestion microscopically by continuously monitoring the event with CCTV. Our methodology explores these three scales for crowd flows, focusing on the microscopic one at the central location of *Place des Terreaux*. The crowd’s movement was notably monitored with strategically placed cameras (Fig. 1) installed by us or by the city of Lyon.

## Methods

### General organization of the data collection campaign

Approximately ten staff members planned and carried out the data collection campaign. The aim was to capture a complete picture of pedestrian motion during the Festival of Lights. For that purpose, we combined various methods targeting different scales. Macroscopically, we inspected the broad patterns of macroscopic crowd flows on the ground around *Place des Terreaux* and recorded the scene with a broad overview. We surveyed passers-by close to the entrance of the square, asking them the following questions:

- ‘How many people were with you?’
- ‘How many children were with you?’
- ‘What was the last screening you attended before the one at Place des Terreaux?’
- ‘What screening do you plan to attend next? (You can answer that you don’t know)’

We strove to limit the selection bias on the the 79 respondents by making no distinction about general or physical appearance and by ensuring that we interviewed a sample from around the square and not limited to a single street.

Mesoscopically, we recruited participants willing to share their GPS data and record how often they bumped into other people.

**Fig. 2:**
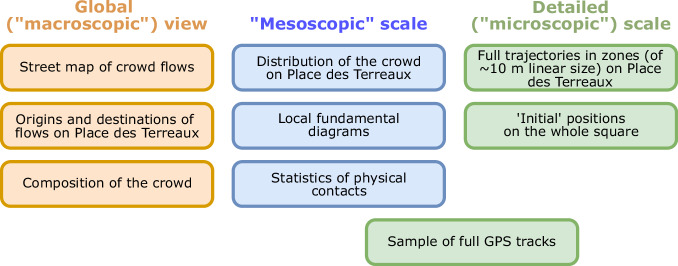
Scales probed in this study and synthesis of the data collected at each scale.

Microscopically, we installed several cameras filming specific zones from the top (with all required authorizations to ensure privacy preservation). Below, we distinguish between *TopView* cameras (labeled 1, 2, 3, 5, 6, 7, 8), filming from a zenithal position, and *LargeView* cameras (labeled 4), recording an even broader view that encompasses the whole square, but with a more tilted (less vertical) angle. An overview of the data collected in this study is presented in Fig. 2.

### GPS data of recruited participants and collision counts

A group of 24 undergraduate and doctoral students from *Institut Lumière Matière* in Lyon were invited to participate in the field study. More precisely, they were asked to follow a general route (from the queue at the entrance to the square, on *Rue du Président Edouard Herriot* to the exit on *Rue Lanterne*, following the flow of the crowd), behave as a standard spectator. Meanwhile, their GPS positions were recorded using the *GeoTracker* application on their smartphones. Although the accuracy of the measurements varied from phone to phone, the trajectories obtained were generally reliable, with a margin of error of around 10 meters on absolute positions. Besides, each participant used a stopwatch on their smartphone to record each time they collided with another pedestrian. Upon synchronization with the GPS data, this method allowed us to precisely determine the time and location of the collisions. Each participant was taught what to consider a collision, which was demonstrated by jostling them hard. Therefore, minor rubbing of the clothing was not recorded. Of the 24 students involved in that experiment, 16 were able to provide us with a detailed list of physical contacts, among which eight could be coupled to GPS data (Dataset^30^, *GPS Data*]). Our efforts to involve on-site spectators in the campaign proved fruitless; none managed to send us GPS data.

### Video processing and pedestrian tracking for the *TopView* cameras

To obtain a finer view of pedestrian trajectories, we deployed lightweight cameras *SJCAM A10* in strategic locations around *Place des Terreaux*. These cameras filmed the scene from a zenith perspective. These cameras were selected for their night vision capabilities and long battery life; three of them, labeled 1, 2, and 3 in Fig. S1 in the Supplementary Material, were placed on the north side of *Place des Terreaux*, protruding from the windows of an apartment *Airbnb* and a restaurant on the second floor. They captured the bidirectional movement of pedestrians below. Another camera, numbered 8, was mounted atop an existing post in the square’s South-East corner to monitor incoming flows; however, its nighttime footage was unusable due to lighting problems. Two additional cameras, numbered 5 and 6, were placed on *Rue Constantine*, one of the main exits after the light show, hanging from the balconies of an *Airbnb* apartment to film the pedestrian egress from above. A final camera numbered 7 was temporarily held at the end of a stick in the South-West corner to provide a closer view of the pedestrian outflow. Overall, the portable *SJCAM A10* cameras recorded nearly 200 GB of video at 30 frames per second.

From the collected footage, pedestrian trajectories were extracted from 10 excerpts (see Table 3 in the Supplementary Material and Dataset^30^, *TopView Trajectories*), using the *PeTrack* software^31,32^. This software is commonly used to automatically detect and track pedestrian heads in controlled experimental settings. The process notably involves calibrating the cameras to match pixel coordinates with real-world coordinates, which is split into two distinct phases: internal and external calibration. Internal calibration corrects the specific optical distortions of each camera lens by determining the optimum parameters to transform a reference pattern (such as a checkerboard) into its recorded image. Then, external calibration yields the conversion between real-world coordinates and pixel coordinates using three successive operations: a rotation and a translation of the reference frame, followed by a projection to move from the camera frame to the screen frame. The parameters of these operations minimize the differences between the known real-world coordinates of objects (here, staff members) positioned at predefined positions (here, at regularly spaced positions on a virtual ‘grid’) and the associated pixel coordinates. Such calibration is only an approximation if the recorded pedestrians are of unequal heights, especially without a stereoscopic camera to reconstruct the scene in three dimensions. Still, the inaccuracy is even lower as the camera is positioned higher and films from a zenithal viewpoint. Here, we expect the maximum uncertainty due to unequal heights to be of order^33^ $\delta h\cdot \frac{D}{H}$, where *H* ≈ 12 m is the altitude of the camera concerning the heads, *D* ≈ 5–10 m is the maximal horizontal stretch between the camera axis and people on the periphery of the scene, and *δh* ≲ 20 cm is the height difference between filmed people and the staff member who served as reference for the calibration; hence below 15 cm in most cases for deployed *SJCAM A10* cameras. The uncertainty in detecting the central point on the head (≲5cm) and the error should be added to this because not all people stand perfectly upright. Furthermore, occlusion phenomena due to the perspective can increase the measurement uncertainty for small pedestrians.

After the calibration step, the *PeTrack* software semi-automatically detects and tracks pedestrians on the videos. Compared to controlled experimental conditions, some difficulties arose. In our case, this task was mainly complicated by the lighting conditions. Thus, pedestrian heads were first detected manually and then tracked from frame to frame using the extended pyramidal iterative Lucas Kanade feature tracker integrated into *PeTrack*. Although this method is surprisingly robust, manual corrections were often required, mainly when the illumination suddenly changed from dark to bright areas.

### Video processing and pedestrian tracking for the *LargeView* camera

In addition to the previously mentioned *TopView* cameras, we have gathered extensive *LargeView* video footage from cameras that offer a bird’s-eye view of the entire square. Two of these cameras are located at *Place Saint-Jean*. Another camera (numbered 4 in Fig. S1 in the Supplementary Material) captures *Place des Terreaux* from the City Hall tower, approximately 48 meters above ground level. Although all recordings are accessible, only the videos from *Place des Terreaux* have been thoroughly analyzed and discussed here.

The expansive view provided by the camera, numbered 4, along with variations in lighting, precludes automated tracking using *PeTrack*, in favor of a largely manual approach. Internal calibration was deemed unnecessary because the camera lens exhibited minimal optical distortion: straight lines in reality appear as straight lines on the video. Complete external calibration was performed by positioning a staff member at predefined evenly spaced points in the square 32 and applying the geometric transformations outlined in Sect. 2. The resulting precision for *absolute* positions, assessed using independently collected positions (either of the same staff member or others), ranged from 10 cm to approximately 2 meters at the farthest end of the square and over 80 meters from the camera horizontally. Given that this inaccuracy is primarily geometric, it is expected that the positions *relative* between pedestrians and their neighbours are significantly more precise.

Initially, we manually identified the positions of all individuals throughout the square at a specific time point, occasionally reviewing video frames to locate pedestrians temporarily obscured from view. This was done after a show cycle, as people began to exit, on Thursday 8 December 2022. Subsequently, we tracked a random sample of approximately 270 individuals over several seconds, with around 100 of them being tracked for a duration of 20 seconds (Dataset^30^, *LargeView Trajectories*], file “LargeView_tracers.txt”). Finally, we focus on a particular area of interest, where opposing flows of people who do not necessarily follow traffic directions meet. The area has a square shape (before the correction of the geometric distortion) and is located near the fountain, a crucial convergence point of high density. Using homemade Python-based software, we manually tracked the trajectories of all observable people in this area over around 30 seconds, at a rate of typically two frames per second. Trajectories were upsampled to 10 Hz by linear interpolation and exported into CSV files (dataset^30^, *LargeView Trajectories*] files “LargeView_zoom_A.txt” and “LargeView_zoom_O.txt”).

It should be noted that, compared to the *TopView* videos, these ones (addressing a more complex flow scenario) are of lower resolution and quality. Combined with the varying illumination, this hindered the detection of some individuals, especially children and shorter people, in specific frames. The datasets may thus exhibit imperfections, such as some individuals escaping detection and occasional swaps between trajectories. However, our subsequent tests have shown that they are accurate to a very large degree and nearly comprehensive (see *Technical validation*).

### Conversion into global coordinates

All trajectories were mapped to global coordinates using the RGF-93 Lambert-93 coordinate reference system (EPSG:2154) for precise global positioning. This transformation involved adjusting the positions of several landmarks visible in the videos and satellite imagery. The geometric shapes and locations of all obstacles in the square, including the fountain, temporary crowd barriers surrounding it, bollards, and Buren columns, were determined via direct measurements, together with satellite imagery and photographs taken on-site; they are provided as an external dataset^30^, *Geometry*].

### Quantitative indicators

Various relevant static and dynamic indicators can be computed using the detailed pedestrian trajectories.

#### Flow rate

The outflow rate during an egress from *Place des Terreaux* was measured by drawing virtual cross-section lines at the two main exits, as shown in Fig. 1. People crossing these lines were counted manually by watching the recorded videos.

#### Density field

To be useful, the extracted microscopic trajectories often need to be smoothed into continuous fields. Specifically, the local density field^34^, denoted as *ρ*(**r**, *t*), is derived by computing the convolution of the microscopic particle density ${\rho }_{\mu }({\bf{r}},t)=\frac{1}{A}{\sum }_{j}\delta ({\bf{r}}-{{\bf{r}}}_{j}(t))$ - where *δ*(⋅) is the Dirac delta function and *A* is the surface area - with a Gaussian kernel ${\phi }_{\xi }({\bf{r}})\propto \exp (-\frac{{{\bf{r}}}^{2}}{2{\xi }^{2}})$ whose integral is normalized to 1, for a chosen half-width *ξ*, viz.:

1 $$\rho ({\bf{r}},t)={\int }_{{\mathcal{A}}}{\rho }_{\mu }\left({\bf{r}}-{\bf{r}}{\prime} \right)\,{\phi }_{\xi }\left({\bf{r}}{\prime} \right)\,{{\rm{d}}}^{2}{\bf{r}}{\prime} $$

For further smoothing, the time dependence can also be coarse-grained by averaging the field over a short time window Δ*t*:

2 $$\bar{\rho }({\bf{r}},t)=\frac{1}{\Delta t}{\int }_{t-\frac{\Delta t}{2}}^{t+\frac{\Delta t}{2}}\rho ({\bf{r}},{t}^{{\prime} })\,{\rm{d}}{t}^{{\prime} }$$

#### Velocity field

Similarly, the microscopic velocities ${{\bf{v}}}_{j}(t)=\frac{1}{\delta t}\cdot [{\widetilde{{\bf{r}}}}_{j}(t+\delta t)-{\widetilde{{\bf{r}}}}_{j}(t)]$, estimated from the vector difference between two positions of pedestrian *j* over a small time interval *δt* (with *δt* = 0.5 or 1 second in this context), can be coarse-grained. The trajectories are initially smoothed using a second-order Butterworth low-pass filter. Trajectories that are too short for effective filtering remain unfiltered. Near the start (*t*_*s*_) and end (*t*_*e*_) of each trajectory, linear interpolation is applied between the raw trajectory **r** and the filtered trajectory $\widetilde{{\bf{r}}}$ to address the Butterworth filter’s limitations when past or future data points are absent. This interpolation is expressed as ${\widetilde{{\bf{r}}}}_{j}(t)\leftarrow \alpha (t){{\bf{r}}}_{j}(t)+[1-\alpha (t)]{\widetilde{{\bf{r}}}}_{j}(t)$, where $\alpha (t)=\max \{{e}^{-(t-{t}_{s})},\,{e}^{-({t}_{e}-t)}\}$. Note that at the start (*t* = *t*_*s*_) and end (*t* = *t*_*e*_) of the trajectory, *α* is close to 1, giving more weight to the original data. This helps preserve the trajectory’s endpoints. In the middle, *α* decreases, giving more weight to the smoothed data. The beginning and end of the raw trajectories are thus preserved, reducing artefacts from filtering. Subsequently, the trajectories are transformed into a velocity field through Gaussian convolution:

3 $${\bf{v}}({\bf{r}},t)=\frac{{\sum }_{j}{{\bf{v}}}_{j}(t)\,{\phi }_{\xi }({\bf{r}}-{\widetilde{{\bf{r}}}}_{j}(t))}{{\sum }_{j}{\phi }_{\xi }({\bf{r}}-{\widetilde{{\bf{r}}}}_{j}(t))}$$

The resulting field exhibits abrupt variations due to many individuals deviating from, or walking counter to, the primary local flow direction. Like the density field, the velocity field can be averaged over a finite time interval Δ*t* to smooth out these variations:

4 $$\bar{{\bf{v}}}({\bf{r}},t)=\frac{1}{\Delta t}{\int }_{t-\frac{\Delta t}{2}}^{t+\frac{\Delta t}{2}}{\bf{v}}({\bf{r}},t{\prime} )\,{\rm{d}}t{\prime} $$

The coarse-grained picture given by the smooth velocity field masks possible counterflows and fluctuations, whose presence can be ascertained by computing a *variance* field:

5 $${{\rm{Var}}}_{{\bf{v}}}({\bf{r}},t)=\frac{{\int }_{t-\frac{\Delta t}{2}}^{t+\frac{\Delta t}{2}}{\sum }_{j}{\phi }_{\xi }({\bf{r}}-{{\bf{r}}}_{j}(t{\prime} )){\parallel {{\bf{v}}}_{j}(t{\prime} )-\bar{{\bf{v}}}({\bf{r}},t{\prime} )\parallel }^{2}\,{\rm{d}}t{\prime} }{{\int }_{t-\frac{\Delta t}{2}}^{t+\frac{\Delta t}{2}}{\sum }_{j}{\phi }_{\xi }({\bf{r}}-{{\bf{r}}}_{j}(t{\prime} ))\,{\rm{d}}t{\prime} }$$

Trajectories of counter-walking pedestrians *j* significantly deviate from the continuous flow, thus exhibiting a large variance relative to the velocity field, denoted as ${{\rm{Var}}}_{{\bf{v}}}^{j}$. More precisely, the variance is computed by averaging the squared difference between pedestrian *j*’s velocity **v**_*j*_(*t*) and the coarse-grained velocity $\bar{{\bf{v}}}({{\bf{r}}}_{j}(t),t)$ at that position, over the entire duration of pedestrian *j*’s trajectory, i.e. ${{\rm{Var}}}_{{\bf{v}}}^{j}=\langle {\parallel {{\bf{v}}}_{j}(t)-\bar{{\bf{v}}}({{\bf{r}}}_{j}(t),t)\parallel }^{2}\rangle$.

## Data Records

The datasets referenced in this study in relation with dense pedestrian dynamics during the 2022 Festival of Lights in Lyon, France, are publicly available and can be accessed via Zenodo^30^. The datasets include macroscopic crowd flows, microscopic individual trajectories, GPS traces, contact data, and statistics on crowd interactions, providing a valuable resource for further research in pedestrian dynamics. In addition, we have released an open online platform that allows users to visualize and plot most of the collected data.

- We have released an open online platform^35^ that allows users to visualize and plot most of the collected data; it can be accessed at https://go.fzj.de/madras-app.
- The survey results are provided in a CSV file, with each line representing a respondent. A README file is included to help interpret the columns.
- The geometry of the square and the (temporary) obstacles are provided as CSV files using the WKT format for geometries.
- Contact data are compiled in a CSV file, detailing the duration, end time, and instances of contact. A README file is included to assist with column interpretation. GPS data for 10 individuals are linked with the contact data. Note that some individuals in the contact data did not provide GPS trajectories.
- Each file is identified by a number, which corresponds to the camera and recording location (see Fig. S1 in the Supplementary Material), and a letter, which indicates the sequence. The videos from which the trajectories were extracted are also available. Trajectories are given in real-world coordinates at a frequency of 10 Hz after all geometric corrections and interpolation have been applied, but without any smoothing or filtering. They are available in both local coordinates (suffix ‘loc’) and absolute coordinates (EPSG:2154) for global location. The time reference is set to Friday 9 December 2022, at 8 pm, with time given in seconds before or after this reference.
- Many more videos were collected than we could analyze. We welcome contributions from volunteers to assist with the tracking efforts. We have made our tracking and camera calibration scripts available to support this.

Descriptive information about the data are available in the Supplementary Material and in ref. ^30^.

## Technical Validation

### Trajectory datasets for the *TopView* recordings

**Fig. 3:**
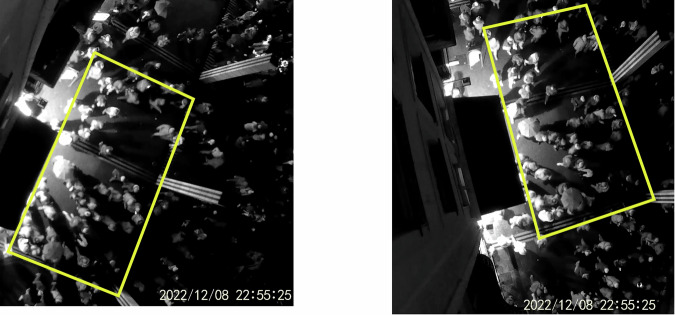
Point of view for the *TopView*_1B (left panel) and *TopView*_2C (right panel) camera videos, which simultaneously captured the same scene from different locations and angles.

**Fig. 4:**
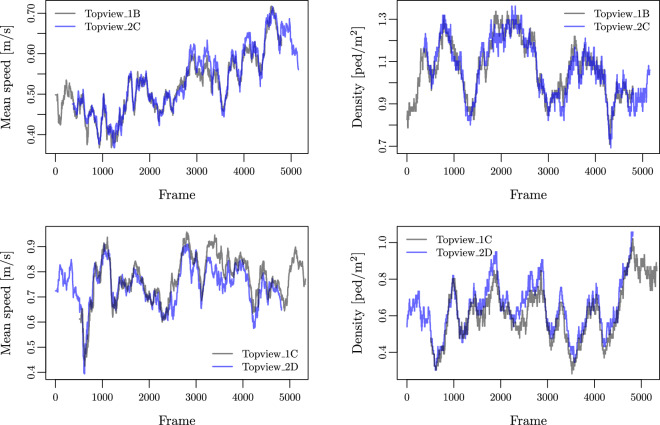
Superposition of the pedestrian mean speed (left panels) and density (right panels) time series for the *TopView*_1B and *TopView*_2C video recordings (upper panels) and for the *TopView*_1C and *TopView*_2D video recordings (lower panels), which capture the same scene from different locations and angles of view, and which partly overlap in time.

All trajectory datasets were visually inspected and manually corrected where necessary. In addition, comparing the overlapping time series of density and mean speed obtained from the *TopView*_1B and *TopView*_2C video recordings, on the one hand, and the *TopView*_1C and *TopView*_2D videos, on the other hand, further validated the results. The fields of view of these cameras are similar (see Fig. 3), and their recordings overlap in time (see Table 3 in the Supplementary Material). We find a relatively small root-mean-square differences (from 5 to 10%) between overlapping sequences, both for the mean speed and for the density (see Table 1 and Fig. 4). Nonetheless, a systematic bias is observed in opposite directions for *TopView*_1B/*TopView*_2C and *TopView*_1C/*TopView*_2D. These biases may be explained by perspective effects and partly biased correction of optical and geometric distortions.

**Table 1:** Root mean square differences (RMSD) between the pedestrian mean speed and density time series for the *TopView*_1B and *TopView*_2C video recordings and the *TopView*_1C and *TopView*_2D video recordings, as shown in Fig. 4.

File	RMSD Mean Speed [m/s]	RMSD Density [ped/m^2^]
*TopView*_1B / *TopView*_2C	0.02	0.05
*TopView*_1C / *TopView*_2D	0.05	0.07

### Trajectory datasets for the *LargeView* recordings

As mentioned above, owing to the lower resolution of the *LargeView* recordings, the quality of the extracted data is not quite as good. Occasionally, we may have failed to detect shorter individuals or swapped intersecting trajectories. Despite these challenges, two staff members (called ‘coders’) independently extracted trajectories from different recordings and then analyzed each other’s work. The coders largely agreed on the extracted data, although there were occasional disagreements or uncertainties regarding some data points. The common trends observed in the density and velocity fields, as shown in Figs. S4-S5 in the Supplementary Material, which correspond to different days of recording, further support the robustness of the presented data.

To further validate the *LargeView* trajectory dataset, possible detection or tracking errors were analyzed jointly by the two coders in a second stage. Errors were categorized as either major or minor. Major errors included omissions of clearly visible pedestrians and the creation of non-existent ones. Minor errors involved misidentifying or possibly swapping pedestrians as well as slightly inaccurate clicks on a pedestrian’s head. The joint analysis led to the detection of 10 major errors (1.3%) out of  ~740 trajectories and 6 minor errors in the *LargeView* Zoom_O dataset, and 3 major errors (0.4%) out of  ~740 trajectories and 5 minor errors in the *LargeView* Zoom_A dataset, over 10 seconds. (All these errors were corrected in the final dataset.)

In order to assess errors on the local densities, two sub-regions of rectangular shape were defined at distinct locations in time and space, each measuring 4 × 6 m^2^, and the two coders separately counted all people in these regions, including flickering appearances who were *likely* to be people, even if this was not certain, in order to get an upper bound. Their respective counts typically differed by less than 10%, and exceeded the number of actually tracked pedestrians by 20% to 30% in the *LargeView* Zoom_O dataset and 9% to 12% in the *LargeView* Zoom_A dataset, depending on the location of the rectangle. This leads to the conclusion that the local densities given by our dataset underestimate the actual densities by *at most* 9% to 30%.

### Mapping to real-world coordinates

To map the pedestrian positions in pixel coordinates to real-world coordinates, calibration using people standing at predefined positions was performed; the distances between the predefined positions were carefully measured on the ground. In the most distant part of the square, the calibration error on the real-world coordinates (but not the *relative* positions) may reach a couple of meters. Then, after conversion, we successfully checked the compatibility of the crowd positions with the geometry of the premises obtained from *Google Earth* data and our independent positioning of obstacles.

### Surveys

Six distinct staff members gave out oral surveys about origins, destinations, and group sizes. In addition to the collected statements, group sizes were also passively observed on the field.

### Respect of privacy

The data that we released on a public repository^30^ do not contain personal or sensitive information. Besides, the video recordings that we collected comply with the GDPR insofar as the recording conditions (from an elevated zenithal viewpoint, at night, without colors) do not allow the identification of passers-by. Finally, the information obtained by means of surveys was aggregated and the results that are presented do not disclose personal details about any of the respondents.

## Supplementary information

Supplementary Information

## Data Availability

Custom code was developed to generate, process, and analyze the datasets presented in this study. Additionally, a Streamlit application enables interactive data exploration and access under specific conditions. The source code is available on GitHub^35^ under the MIT license distribution, and modification with proper attribution. The repository includes:• The scripts and notebooks used for data collection, preprocessing, analysis, and visualization.• The Streamlit application code, along with instructions for installation and deployment^35^.• A requirements.txt file specifying the versions of the software and libraries used to ensure compatibility. Essential software versions include PedPy 1.0.2^36^ as a backend for speed, density and flow calculations. The repository also includes comprehensive documentation that details the specific variables, parameters, and settings used to facilitate the reproduction of the results and analyses presented in this study. This encompasses, but is not limited to, parameters for data filtering and the statistical analysis methods applied.
