# Supplementary material for: Dense Crowd Dynamics and Pedestrian Trajectories: A Multiscale Field Dataset from the Festival of Lights in Lyon
Source: Sci Data. 2025 Apr 30;12:718. doi: 10.1038/s41597-025-04732-3 (PMC12043992; doi:10.1038/s41597-025-04732-3)
Supplement: Supplementary file 1 — Supplementary Information [file 41597_2025_4732_MOESM1_ESM.pdf]

Dense Crowd Dynamics and Pedestrian Trajectories: A Multiscale Field Dataset from the Festival of Lights in Lyon — Supplementary Material

Oscar Dufour[1,*], Huu-Tu Dang[2], Jakob Cordes[3,5], Raphael Korbmacher[4], Mohcine Chraibi[3,*], Benoit Gaudou[2,*], Alexandre Nicolas[1,*], and Antoine Tordeux[4,*]

[1]Universite Claude Bernard Lyon 1, CNRS, Institut Lumière Matière, UMR5306, F-69100, Villeurbanne, France
[2]UMR 5505 IRIT, Université Toulouse Capitole, Toulouse, France
[3]Institute of Advanced Simulation, Forschungszentrum Jülich GmbH, Jülich, Germany
[4]Fakultät für Maschinenbau und Sicherheitstechnik, Bergische Universität Wuppertal, Wuppertal, Germany
[5]Institut für Theoretische Physik, Universität zu Köln, Köln, Germany
[*]Corresponding authors: Oscar Dufour (oscar.dufour@univ-lyon1.fr), Mohcine Chraibi (m.chraibi@fz-juelich.de), Benoit Gaudou (benoit.gaudou@ut-capitole.fr), Alexandre Nicolas (alexandre.nicolas@cnrs.fr), Antoine Tordeux (tordeux@uni-wuppertal.de)

A Size of the crowd and flow directions

Place des Terreaux is centrally located in Lyon and is a key attraction during the Festival of Lights. Pedestrian traffic around the square is regulated: spectators enter from the South-East via *Rue du Président Edouard Herriot*, typically remain in the square for the duration of one show (6 minutes and 30 seconds), sometimes two, and then exit either to the South-West via *Rue Constantine* or to the North-West via *Rue d'Algérie*, as illustrated in Fig. S1b.

Field surveys conducted around 11 pm on Friday revealed that most spectators had previously seen light animations just south of the square. Upon entering *Place des Terreaux*, many were uncertain about their next destination or planned to head home. These large-scale origin-destination flows are depicted in Fig. S1a. Spectators usually belong to social groups of two to four people; larger groups, up to ten people (and above), also exist, but become less frequent as the group size increases. Most groups do not include children, although groups with one or two children were observed several times, as shown in Fig. S18.

The entrance to *Place des Terreaux* is managed by gatekeepers who ensure the square does not exceed approximately two-thirds of its maximum capacity. They restrict access by closing a barrier before the start of each show, leading to a queue of people standing on *Rue du Président Edouard Herriot* that can stretch over several blocks. During a light show, the number of people in *Place des Terreaux* can significantly exceed 4 000, according to our manual detections and counts on one snapshot extracted from the videos (refer to Table 1).

This attendance number is corroborated by the cumulative pedestrian outflow measured at the two exits, *Rue Constantine* and *Rue Paul Chenavard*, at the end of a show, from 9:38 pm to 9:45 pm on December 9, 2022 (see Sec. 2); the total evacuation time was approximately 6.5 minutes. The evolution of pedestrian outflows over time is shown in Fig. S2. It displays the raw, instantaneous values (depicted by dashed black lines) and the smoother curves (solid black line) obtained by applying a Gaussian filter with a kernel standard deviation of 2.0. The maximum outflow exceeded 11 ped/s on *Rue Constantine* and nearly reached 10 ped/s on *Rue Paul Chenavard*. In total, 3833 pedestrians were counted, with 1803 on *Rue Constantine* and 2030 on *Rue Paul Chenavard*.

Around 4 000 people on the whole square correspond to a global density below one pedestrian per square meter. However, as we will observe, the global average is not particularly insightful due to significant spatial heterogeneity. It is important to note that both counting methods tend to underestimate the actual numbers. Not all individuals are visible on a snapshot, while the estimate based on the outflows discards the spectators who did not leave the square after the show. Besides, despite the regulated inflow, variations in the number of attendees occur during each cycle. However, quite remarkably, visual inspection of the entrance and egress flows points to a high degree of regularity of the flows across cycles. In particular, obstacles and prohibited areas (marked in blue in Fig. S3) visibly alter the flow pattern, creating a confluence zone between the fountain and the northern building, along with congestion points on the opposite side of the fountain. In contrast, the crowd moves almost freely along the southern side, where projections are displayed on a building during the show, thus making this viewpoint less attractive to spectators (see Fig. 2).

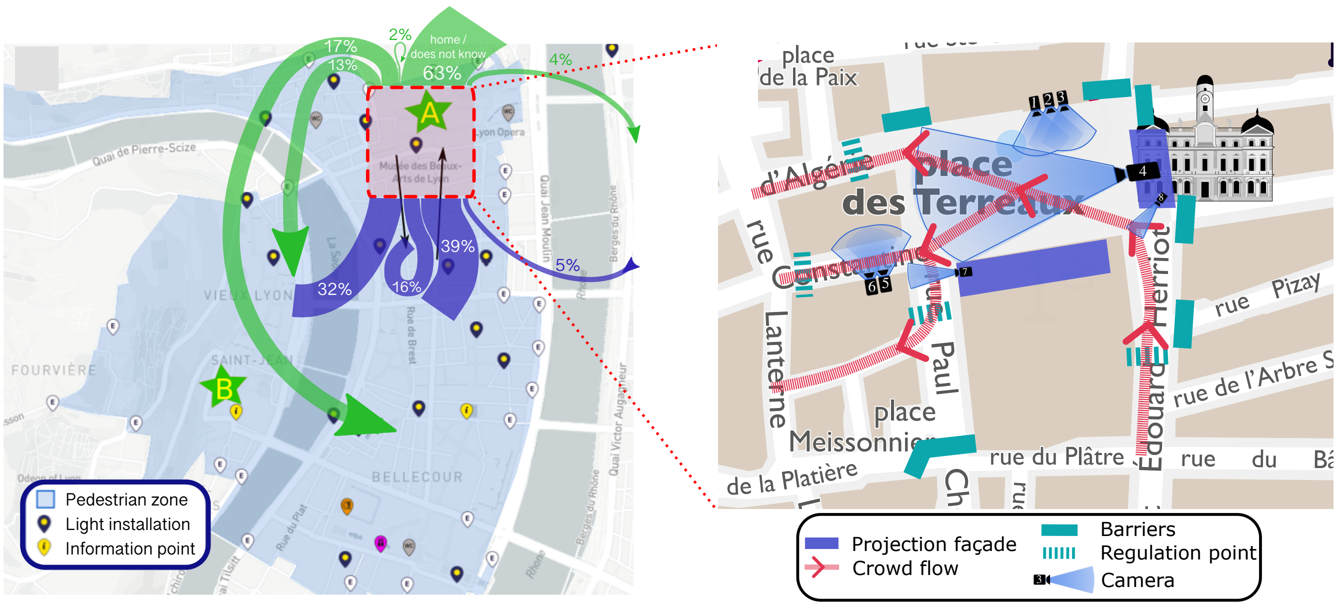

Figure S1. Maps of the macroscopic crowd flows during the Festival of Lights. **Left panel:** Pedestrian zone adapted from the official map[1] indicating the distribution of origins (in blue) and destinations (in green) of around 300 people, just before and just after the show on *Place des Terreaux*, obtained by surveying 79 passers-by around 11 pm on Friday 9 December 2022. **Right panel:** Local map showing the imposed flow directions around *Place des Terreaux*. All figures are oriented to the North unless otherwise shown in the figure.

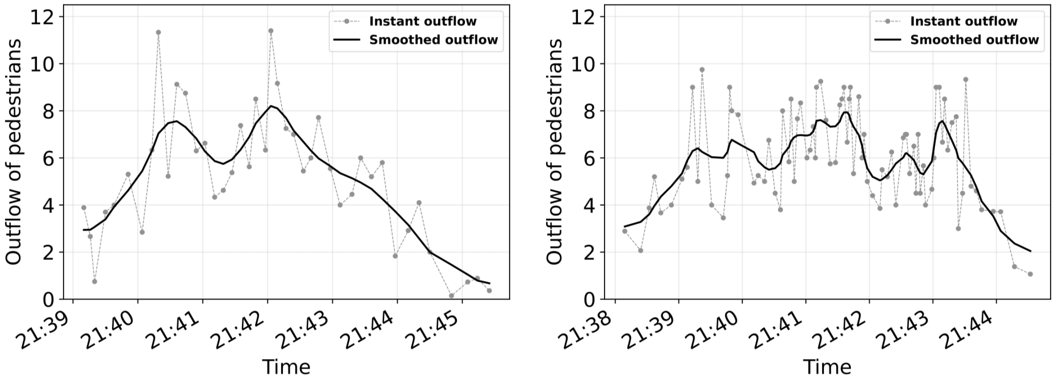

Figure S2. Pedestrian outflows measured during one of the periodic egresses from *Place des Terreaux* on December 10, 2022. **Left panel:** Rue Constantine. **Right panel:** Rue Paul Chenavard.

B Global view of the flow patterns on *Place des Terreaux*

To better understand the distribution of the crowd and the flow patterns during the evacuation of the square after a show, we analyzed the *LargeView* video recordings. From a snapshot captured at the start of the repeated evacuation process, we manually extracted the positions of all visible heads in the crowd. These positions are represented as small red disks in Fig. S3, left panel, along with the accessible geometry of the square. The heterogeneous spatial distribution is manifest and becomes even more pronounced in the corresponding density field shown in Fig. S3, right panel, where local densities range from nearly 0 ped/m^2 to 4 ped/m^2. The trajectories of approximately 100 randomly sampled pedestrians tracked over about 20 seconds and shown in Fig. S3, left panel (also see Supplementary Video[2]), also exhibit marked heterogeneity, with a significant portion of pedestrians halted and some moving counter to the flow. To get a broader perspective on this heterogeneity, we sampled 270 people in the crowd (including the 100 already tracked pedestrians) and measured their initial velocities (over only a few seconds) soon after they were instructed to exit the square at the end of a light show, as shown in Fig. S3, right panel (Dataset [3, *LargeView Trajectories*]). The resulting fundamental diagrams are presented in Fig. S15.

General flow patterns are discernible during the evacuations of *Place des Terreaux*. Most people head West, towards the

two main exits (located at the top of the picture in Fig. S3). Between the fountain and the northern building (to the right of the picture), the flows (mainly, but not exclusively, directed to the West) go through a zone of convergence, which will be probed in greater detail below. On the road along the southern building, most trajectories stretch linearly, from East to West, in almost free-flowing conditions. In contrast, trajectories observed closer to the fountain are significantly shorter over the same time interval, pointing to congestion and more diverse behaviors, with many people at a standstill.

File	Date [UTC+1]	Duration	# Initial Positions	# Long (~ 20 s) Trajectories / All	Mean / Median Speed [m/s]
LargeView	December 8, 2022 20:13	20 s	4081	114 / 277	0.44 / 0.36

Table 1. Basic statistics for the *LargeView* dataset [3, *LargeView Trajectories*] inspected over the whole square.

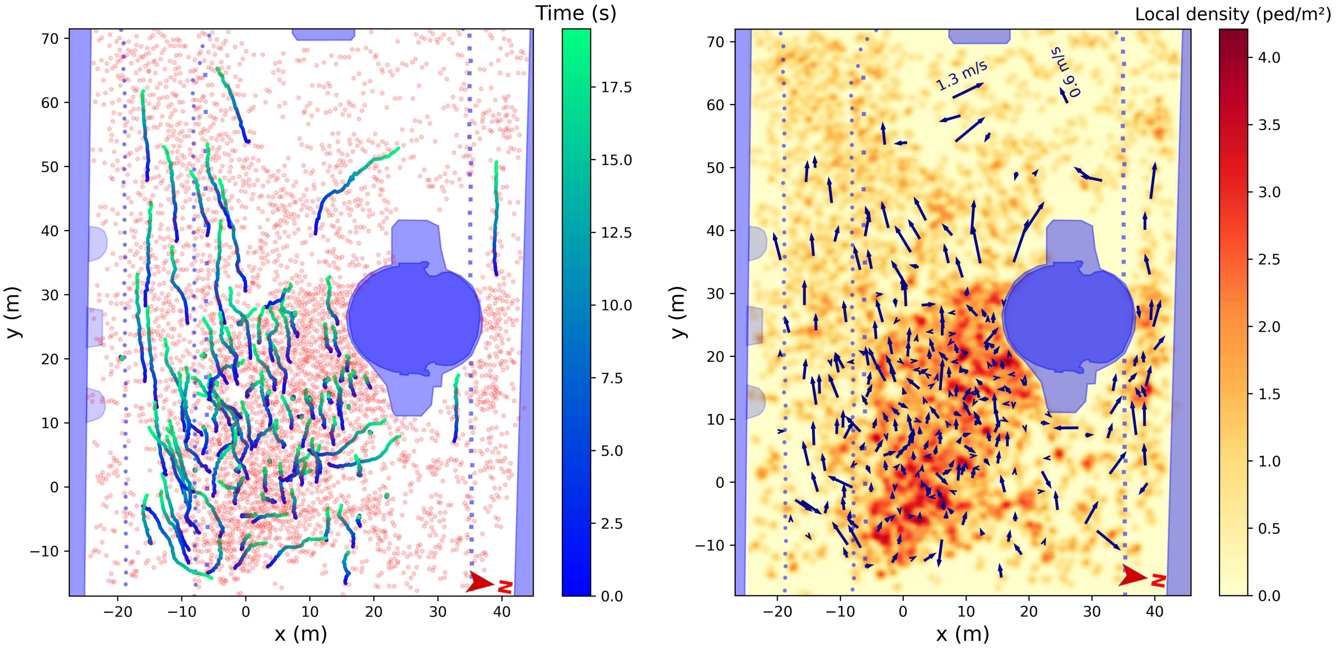

Figure S3. Comprehensive overview of the crowd's positions and dynamics on *Place des Terreaux*. **Left Panel:** Trajectories of approximately 100 pedestrians tracked over a span of 20 seconds. Small red disks mark the initial positions of all individuals in the square. Various obstacles, such as fountains, buildings, bollards, and barriers, are shaded in blue (Dataset [3, *Geometry*]). **Right Panel:** Initial velocities of around 270 pedestrians, computed with a time step of $\Delta t = 1\,\mathrm{s}$. The background features a heat map representing the initial density field, computed using a Gaussian kernel with a half-width of $\sigma = 0.5\,\mathrm{m}$.

Complex flow in a region of high density near the centre of the square

Let us delve deeper into the pedestrian flows by examining the central zone, highlighted by the pink rounded square in Fig. 2 (also see the related statistics in Table 2). This area, measuring 15 meters by 25 meters, exhibited complex patterns and high densities during two distinct periodic egresses. We tracked all pedestrians within this zone semi-manually, limited by the video resolution and occasional occlusions (see Sec. 2 for details). The extracted trajectories can be used to construct and animate a 'digital twin' of the crowd. This animation[4] does not account for the heterogeneous sizes of pedestrians, representing all agents as standard adults, nor does it consider their social relationships. At first glance, there is a noticeable overall flow towards the West (the top of the image). However, the flow pattern is non-uniform, featuring counter-flows, individuals squeezing through the crowd, and others moving slowly.

The complex features that obscure the general characteristics can be simplified by coarse-graining trajectories into smooth density and velocity fields, as illustrated in the left panels of Fig. S4 and Fig. S5. These smooth fields reveal noticeable density heterogeneities, but the flow pattern is more streamlined: most velocity vectors are aligned, directed towards the top

of the figure, and rarely exceed half a meter per second. A slight tendency to navigate around densely populated areas is still observable. These coarse-grained fields can be interpreted as the underlying base flow. On top of this base flow, the variability of trajectories can be reintroduced by calculating local velocity variances, as shown in the right panels of Fig. S4 and Fig. S5, highlighting trajectories that significantly deviate from the base flow (see Sec. 2). This distinction between a smooth, streamlined base flow and counter-walking agents may be beneficial from a modelling perspective, allowing for advancements beyond the homogeneous flows predicted by macroscopic models.

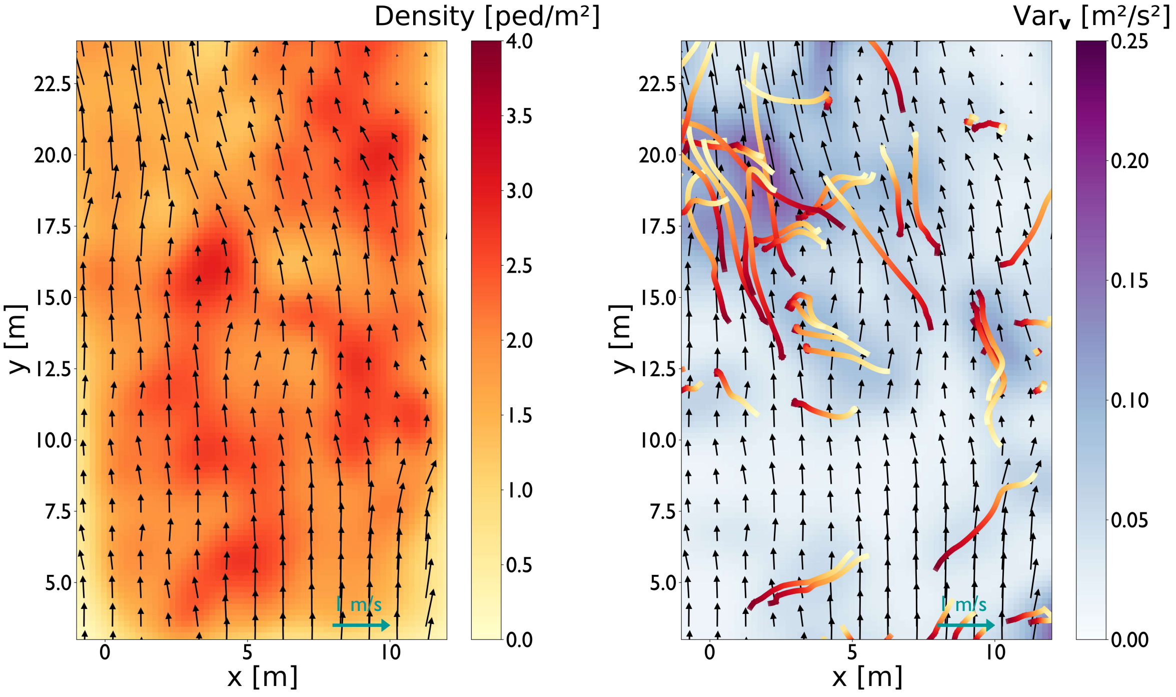

Figure S4. Continuous description of the complex flow at the center of the *Place des Terreaux* at 20:15 on December 8th, recorded with a *LargeView* camera (same orientation as Fig. S3). **Left panel:** Local density field averaged over the time window of 10 seconds in our local coordinate system. **Right panel:** Velocity variance field $\text{Var}_\mathbf{v}(\mathbf{r},t)$ averaged over the same time window. The displayed trajectories (colored from red to yellow as time moves on) are those of 'counter-walking' pedestrians, i.e., those who significantly deviate from the continuous velocity field by $\text{Var}^i_\mathbf{v} \geqslant 0.9\ \text{m}^2/\text{s}^2$. The arrows represent the continuous velocity field over the same time window of 10 s. All fields have been smoothed with a characteristic lengthscale $\xi = 0.75\,\text{m}$.

**File**	**Start** [UTC+1]	**Duration**	**# trajectories**	**Mean density** [ped/m^2]	**Mean / median speed** [m/s]	**Median / Mean trajectory duration** [s]
LargeView Zoom_A	December 8, 2022 20:16	20 s	740	1.92	0.30 / 0.29	17.50 / 17.98
LargeView Zoom_O	December 9, 2022 21:05	45 s	726	1.85	0.29 / 0.27	33.21 / 41.7

Table 2. Basic statistics for the exhaustive trajectory dataset [3, *LargeView Trajectories*] extracted from an area of interest in the *LargeView* videos.

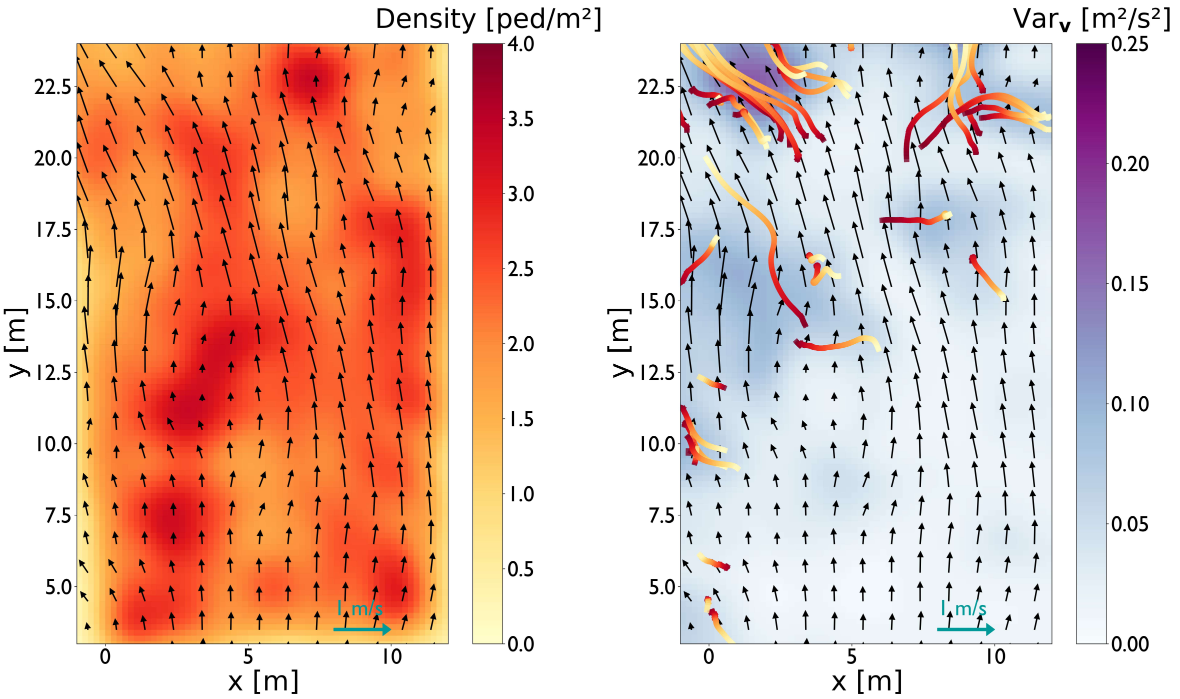

Figure S5. Continuous description of the complex flow at the center of the *Place des Terreaux* at 21:05 on December 9th, recorded with a *LargeView* camera. Refer to Fig. S4 for the rest of the caption.

C Unidirectional and bidirectional flow at diverse densities along the northern building

The flow pattern along the northern building of *Place des Terreaux* (see Fig. 2) looks less complex compared to the preceding central region. In particular, there is a primary East-West direction. Besides, for these trajectories, the resolution and orientation of the camera (*TopView* camera 2) afford higher-quality microscopic data. Notwithstanding this apparent relative simplicity, we will see that the crowd flows depart from their idealizations as unidirectional and bidirectional flows in controlled experiments, but to different extents. Corresponding fundamental diagrams are shown in Fig. S16.

**File**	**Start** [UTC+1]	**End** [UTC+1]	**# trajectories**	**Mean density** [ped/m^2]	**Mean speed** [m/s]	**Median / Mean trajectory duration** [s]
*TopView*_1A	22:40:45	22:44:15	965	1.13	0.57	13.23 / 12.27
*TopView*_1B	22:55:06	22:57:46	685	1.07	0.52	11.76 / 12.64
*TopView*_1C	23:10:33	23:13:58	673	0.65	0.78	9.2 / 9.03
*TopView*_2A	21:26:27	21:29:07	711	1.58	0.41	19.1 / 18.11
*TopView*_2B	21:40:39	21:43:25	612	1.11	0.58	11.17 / 13.18
*TopView*_2C	22:55:18	22:58:20	693	1.06	0.54	11.96 / 12.94
*TopView*_2D	23:10:16	23:12:57	529	0.64	0.75	8.83 / 8.96
*TopView*_2E	23:24:59	23:26:11	218	0.57	0.75	8.83 / 8.63
*TopView*_2F	23:54:59	23:56:30	183	0.37	0.95	6.8 / 6.92
		Total	5269	0.96	0.63	10.63 / 12.22

Table 3. Basic statistics for the exhaustive trajectory datasets [3, *TopView Trajectories*] extracted from *TopView* videos over different time intervals at the right part of the *Place des Terreaux* (see Fig. 2).

Unidirectional pedestrian flow (*TopView*_2B)

In the *TopView*_2B video recording, unidirectional flow prevails. Among the 612 collected trajectories, 496 pedestrians move from right to left, 60 from left to right, 46 from bottom to top, and 10 from top to bottom. In the left panel of Fig. S6, trajectories are color-coded based on their entry and exit points: green for entry on the left and exit on the right, grey for entry on the right and exit on the left, red for entry from the bottom and exit at the top, and blue for entry from the top and exit at the bottom. Despite the prevailing unidirectionality, it is noteworthy that the streamlines are not strictly parallel in this essentially unconstrained geometry, even when focusing solely on the grey trajectories. Examining the density and speed profiles, computed using Gaussian-kernel filters (see Eq. (3) and the documentation in[4] and[5]), Fig. S6 (right panel) reveals that they are relatively uniform, except for a few pedestrians standing in the upper left of the scene, which results in density peaks.

Finally, Fig. S7 presents a time series of various global indicators. The cumulative inflows from the four directions confirm the dominance of pedestrians moving to the right. Interestingly, there is a noticeable evolution of density over time. From $t = 30$ s onward, the density steadily increases from 0.4 to 2.2 ped/m^2, as observed in the snapshots of Fig. S8. The average speed in the area mirrors this trend, decreasing steadily from about 1 to 0.3 m/s. These trends align with expectations based on existing literature on unidirectional flow in controlled experiments. The reduced average speed at low density can be ascribed to static individuals and the presence of social groups, which are known to reduce walking speed[6].

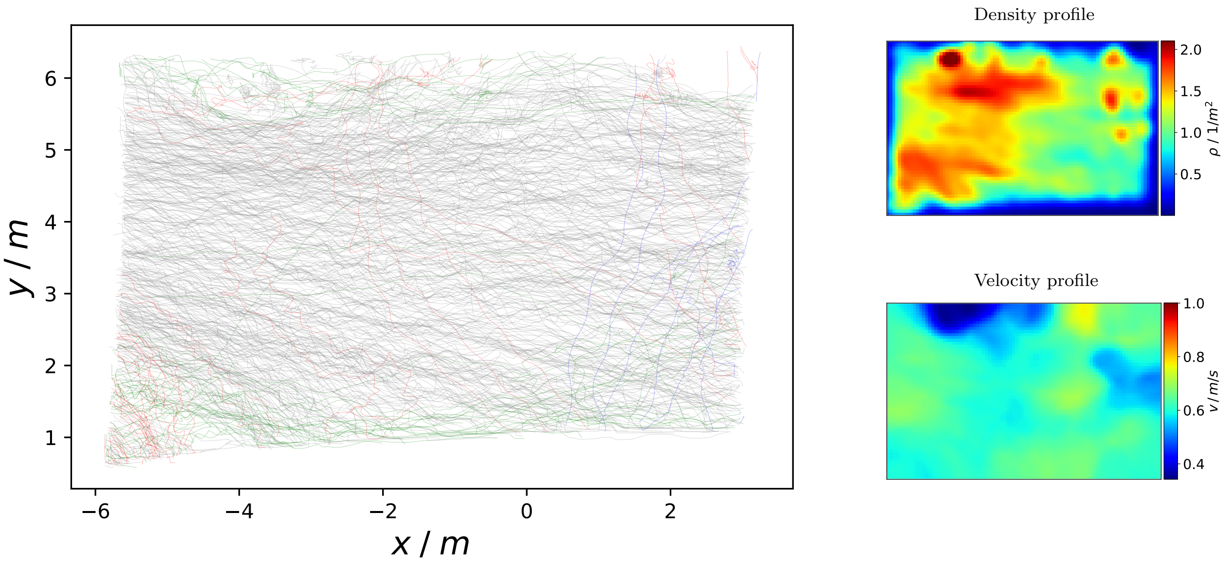

Figure S6. Trajectories **(left panel)** with density and speed profiles **(right panels)** for the *TopView*_2B video recording, showing predominantly unidirectional pedestrian dynamics.

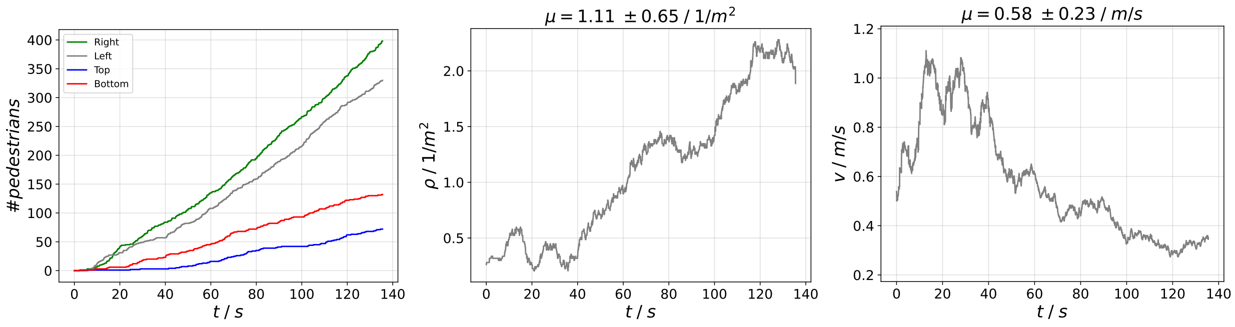

Figure S7. Cumulative flow (**left panel**), density (**middle panel**), and mean-speed time-series (**right panel**) for the *TopView*_2B video recording. The density increases over time for this video recording while the speed decreases.

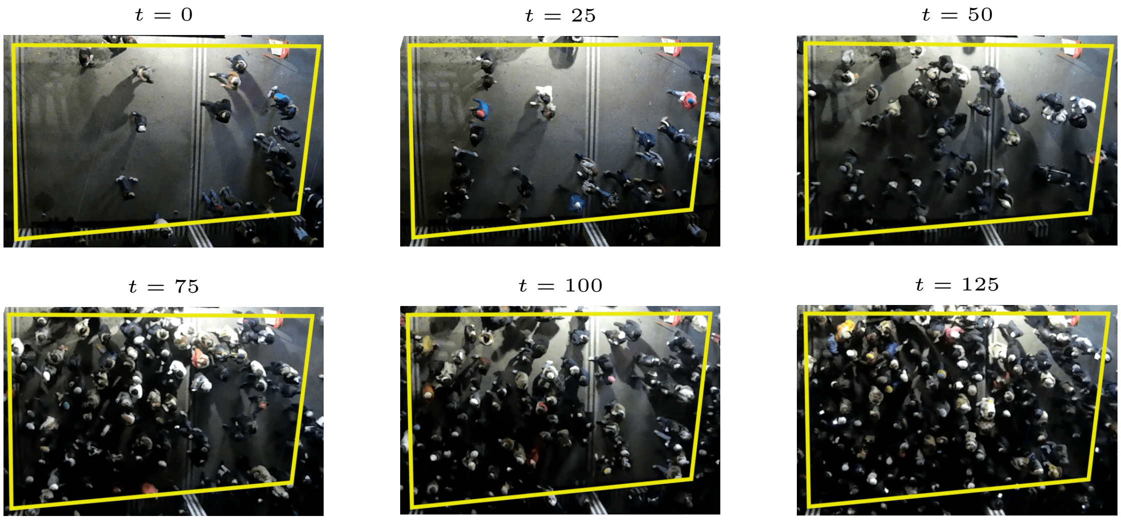

Figure S8. Snapshots of the *TopView*_2B video recording at different times.

Unidirectional flow with standing pedestrians as obstacles (*TopView*_2C)

The sequence *TopView*_2C, in the same zone, features an additional perturbation: a group of pedestrians standing in the upper left part of the scene. Out of the 693 trajectories, 603 walk to the left, 68 to the right, 15 up and seven down; the flow is thus predominantly unidirectional again. However, the static group has a conspicuous effect on the trajectories, forcing other pedestrians to go around it and causing congestion in the dynamics (see Fig. S9, left panel). The density and speed profiles show two congested queues with reduced speed among the standing group (see Fig. S9, right panels). The state is stationary in time, with a global density fluctuating between 0.8 and 1.3 ped/m^2 and a mean speed between 0.4 and 0.7 m/s (see Fig. S10). This is confirmed by the snapshots in Fig. S11, which show similar crowding situations.

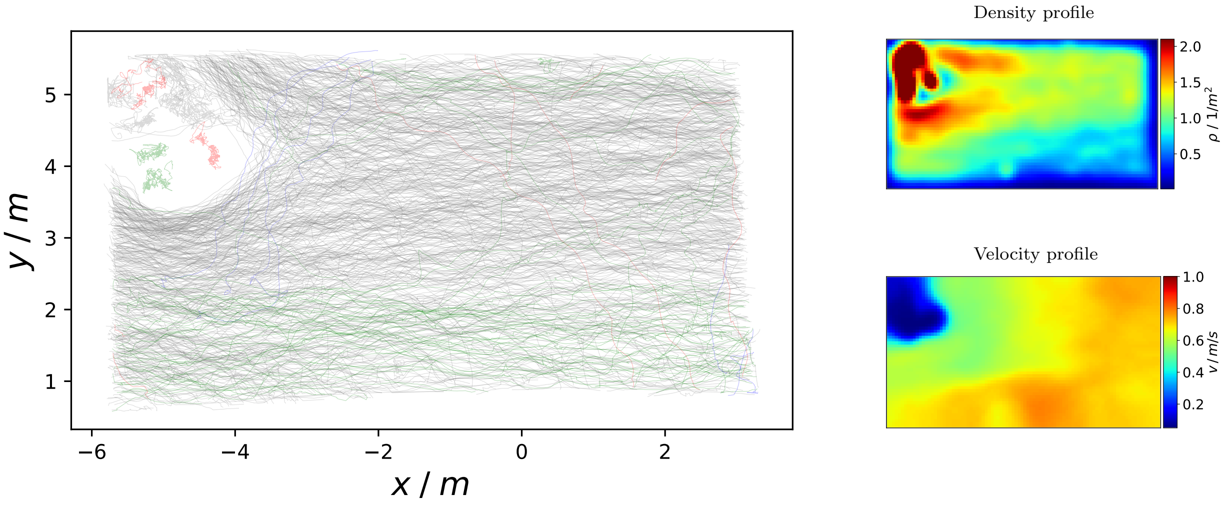

Figure S9. Trajectories **(left panel)** with density and speed profiles **(right panels)** for the *TopView*_2C video recording. The pedestrian dynamics are predominantly unidirectional, while the scene includes standing pedestrians in the upper left corner, initiating avoidance behaviour and queuing.

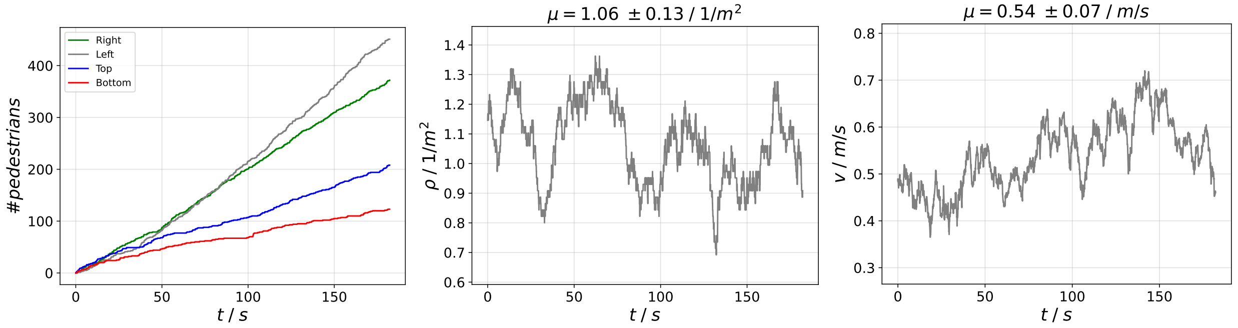

Figure S10. Cumulative flow (**left panel**), density (**middle panel**), and mean-speed time-series (**right panel**) for the *TopView*_2C video recording. The situation is relatively stationary in time.

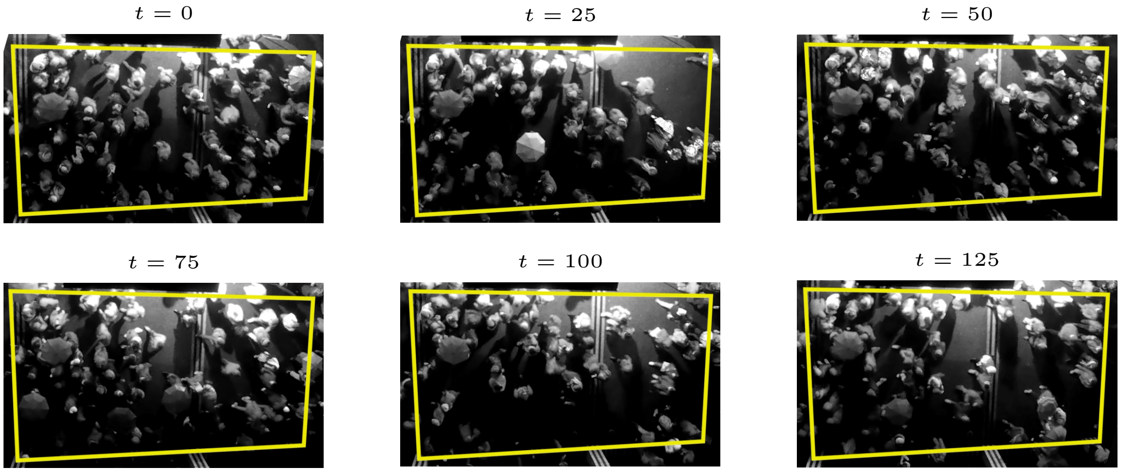

Figure S11. Snapshots of the *TopView*_2C video recording at different times.

Unbalanced birectional flow (*TopView*_2D)

The last recording that we analyze, Topview_2D, contains 529 pedestrian trajectories, including 396 trajectories to the left, 109 to the right, 13 upward and 13 downward (see Figure S12, left panel). The counter-walking pedestrians can no longer be neglected. They generate a substantial counterflow with lane formation by direction, separated by a group of standing pedestrians in the centre right of the scene. Accordingly, we are dealing with an unbalanced (75%:21%) bidirectional flow. The density and speed profiles are relatively homogeneous, although the flow to the left is slightly more congested (see Figure S12, right panels). Again, the state is relatively stationary in time, with a global density fluctuating in between 0.4 and 1 ped/m^2 and a mean speed between 0.5 and 0.9 m/s (see Figure S13). The snapshots show that the crowd is sparser than in the previously presented video recordings (see Figure S14).

D Fundamental diagram

The fundamental diagram is obtained by relating the instantaneous pedestrian speeds $\|\mathbf{v}_j(t)\|$ to the local density ρ (binned in cells of linear size 0.25 m and duration 0.5 s). For more information on defining quantitative indicators, refer to the documentation of the MADRAS-Streamlit[4] and PedPy[5] libraries.

Plotting these results in terms of initial speed against local density provides the fundamental diagram presented in Fig. S15 (left). Compared to conventional fundamental diagrams[7], largely obtained in controlled settings, it exhibits much more scatter. In particular, while the largest speeds observed at a given density form an envelope curve broadly compatible with Weidmann's empirical formula[8] $v(\rho) = v_0 \cdot \left[1 - \exp\left(-\gamma \cdot (\frac{1}{\rho} - \frac{1}{\rho_{\max}})\right)\right]$, many speed data points fall between 0 m/s and this curve. They correspond to people strolling, sometimes because they are moving along (and possibly chatting) with a social group or who are halted. Interestingly, at a superficial level, this observation mirrors the doubts on the uniqueness (i.e., bijectivity) of the

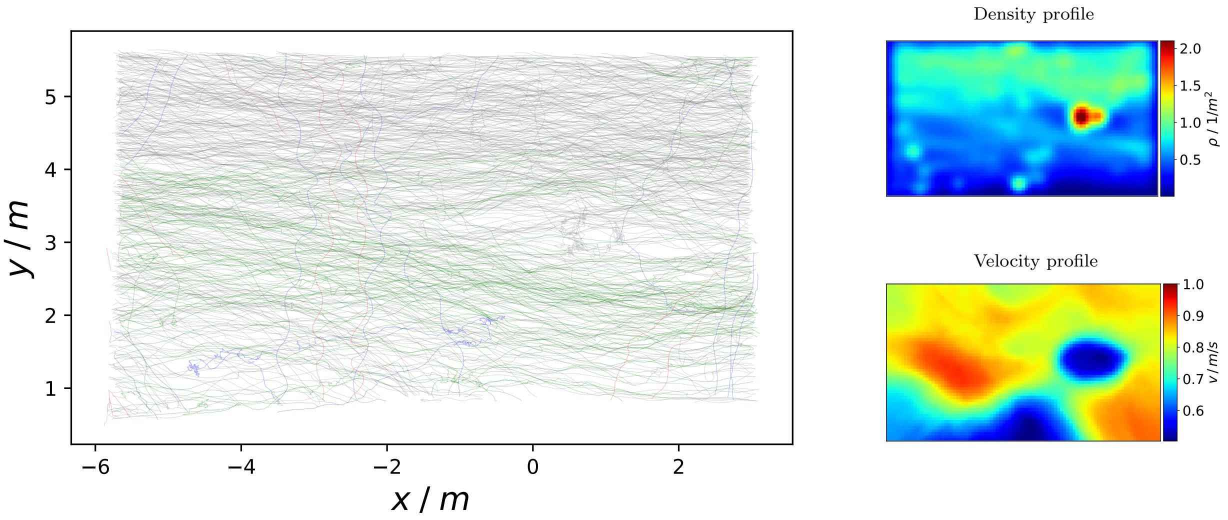

Figure S12. Trajectories **(left panel)** with density and speed profiles **(right panels)** for the *TopView*_2D video recording, showing mainly counterflow pedestrian dynamics with lane formation.

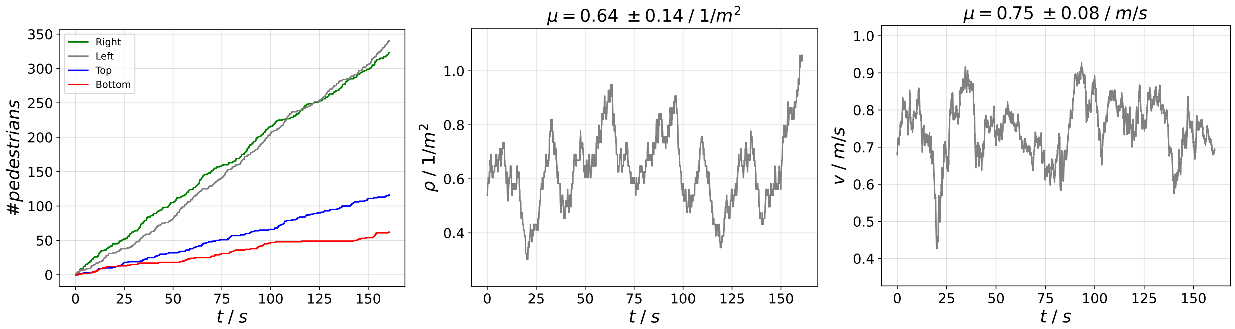

Figure S13. Cumulative flow (**left panel**), density (**middle panel**), and mean-speed time-series (**right panel**) for the *TopView*_2D video recording. The situation is relatively stationary in time.

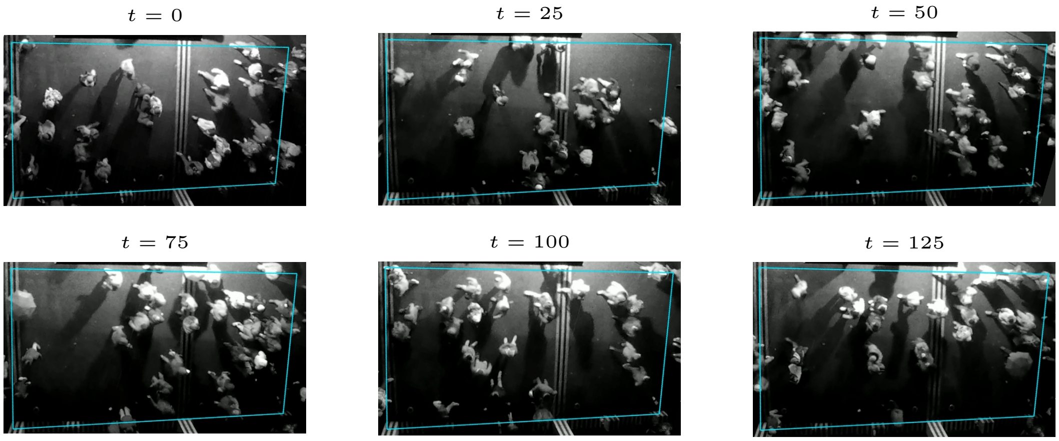

Figure S14. Snapshots of the *TopView*_2D video recording at different times.

mapping between speed and density in vehicular traffic and the arguments in favour of a fundamental diagram spanning a two-dimensional region of the plane[9]. Note that the fundamental diagram binned into time intervals is essentially similar to that obtained for a sample of pedestrians across the entire square; the maximum speed observed at a given density exhibits a downward trend with increasing density, but all speeds below this upper bound are represented.

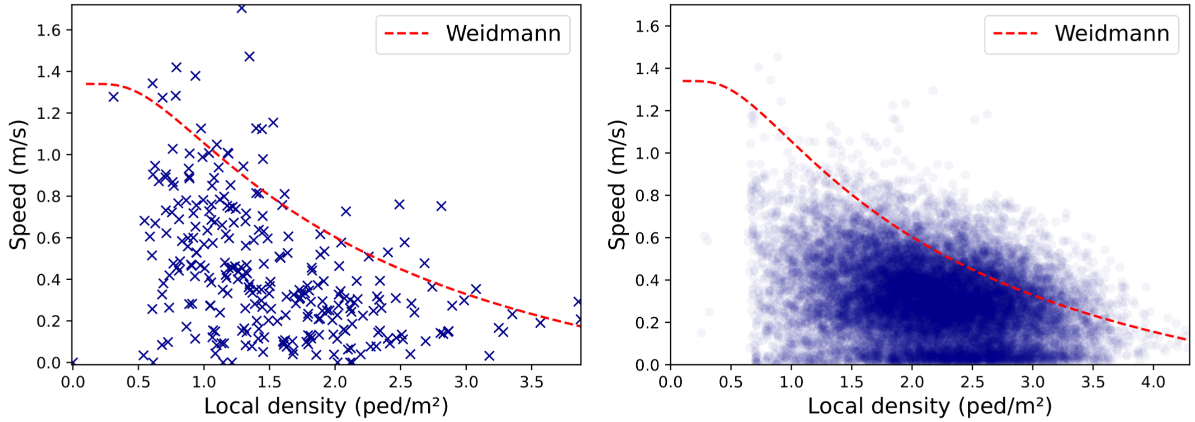

Figure S15. Fundamental diagrams relating pedestrian speed to the local density. The **left panel** depicts this relationship across the entire *Place des Terreaux*, using a sample of initial velocities as shown in Fig. 5 (right panel). The **right panel** focuses on a central region of the square, where pedestrians were exhaustively tracked for approximately 20 seconds (see Fig. 6). Dashed red lines: Weidmann's empirical formula $v(\rho) = v_0 \cdot \left[1 - \exp\left(-\gamma \cdot \left(\frac{1}{\rho} - \frac{1}{\rho_{\max}}\right)\right)\right]$ with parameters $v_0 = 1.34\,\mathrm{m/s}$, $\gamma = 1.9\,\mathrm{m}^{-2}$, and $\rho_{\max} = 5.4\,\mathrm{ped/m}^2$.

The fundamental diagram for the *TopView* trajectories is presented in Fig. S16. Here we averages the speed of the trajectories and the *global* density level in one-second intervals. This relationship displays much less scatter than the measurements at the center of *Place des Terreaux* (see Fig. S15). Furthermore, Table 3 provides basic statistics for each of the nine trajectory datasets, with three sequences selected explicitly for detailed analysis.

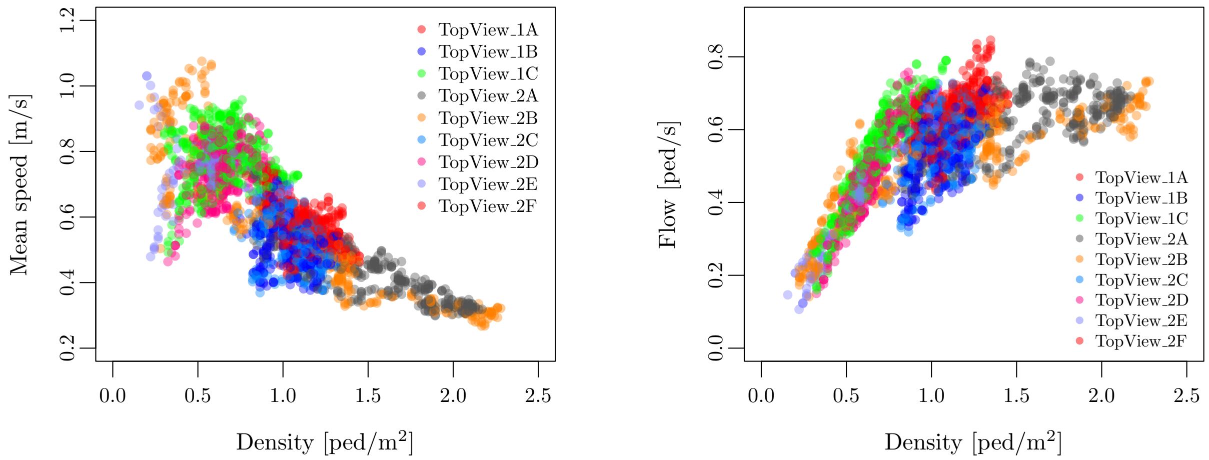

Figure S16. Fundamental diagram obtained by averaging the speed of the trajectories and the global density level in the scene over one-second time steps, for the *TopView* datasets. **Left panel:** Mean speed-density relationship. **Right panel:** Flow-density relationship. Here the flow is determined as the product of the density and the mean speed.

E GPS traces and statistics of physical contacts

The GPS trajectories collected from informed participants (Dataset [3, *GPS Data*]), as depicted in Fig. S17, illustrate potential routes from the square's entrance to its exit and beyond. By synchronizing these trajectories with the reported times of pushes and strong contacts, we identified where these interactions occurred and marked their locations as stars on Fig. S17. Conspicuously, the total numbers of reported pushes vary significantly among participants, ranging from nearly zero to approximately 100 throughout the trajectory, as noticeable in Fig. S17. These variations highlight the heterogeneity of the crowd packing, the diversity of individual behaviours, and, plausibly, different appraisals of what should be counted as a push. Still, the order of magnitude of the frequency of strong contacts questions the collision-free navigation hypothesis at the heart of some models based on velocity obstacles[10, 11], but also, at the other pole, the strong role played by contact forces at densities below 4 ped/m^2 in other (typically force-based) force-based models.

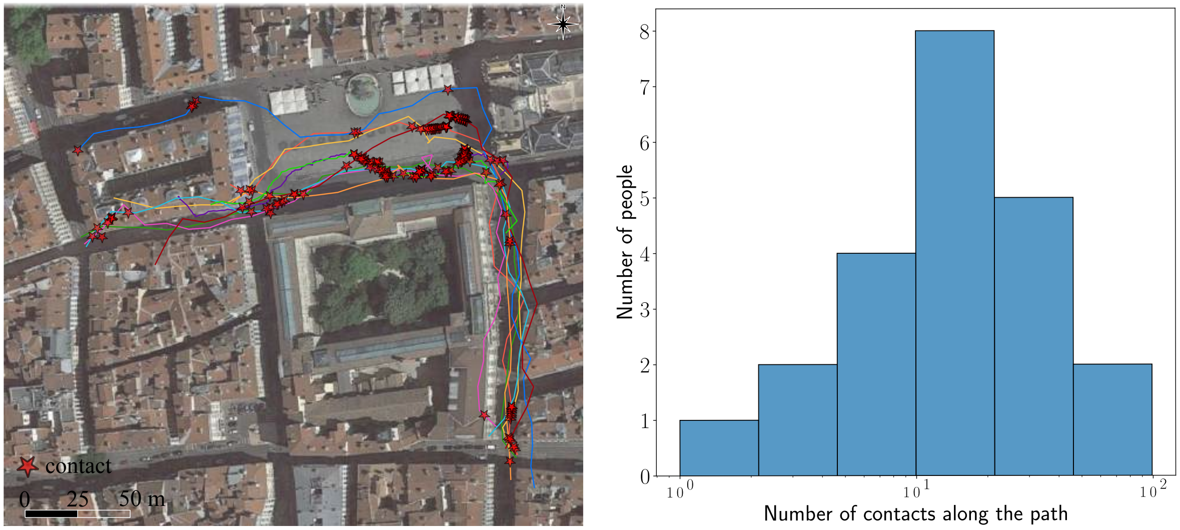

Figure S17. Left panel: Ten GPS tracks are paired with contact data, with each contact marked by a red star. Clusters of red stars appear at the entrance and exit of *Place des Terreaux*. Note that the satellite view timing does not match the data, and some GPS tracks overlap buildings due to varying GPS precision across devices, providing a general idea of contact locations. **Right panel:** A histogram showing the distribution of contact counts along the path on a logarithmic scale, incorporating data from the left panel and additional entries without GPS tracks.

F Identification of singular qualitative phenomena

This section highlights key features passively observed during the real-world Festival of Lights, a complex scenario that significantly diverges from controlled settings. Many of these features are due to the multidirectionality of the flow or, more generally, the fact that the pedestrians composing the crowd have different goals.

These features notably include:

- temporarily static groups of people around which passing pedestrians are forced to circumnavigate
- marked spatial variations of the density
- the serpentine motion of groups of people following each other through the crowd, thus moving like snakes (dubbed 'crossing channels' in a controlled experimental study[12] of crossing)
- various non-standard pedestrians: people pushing a pushchair, pulling a piece of luggage, etc.
- a complex geometry of the premises that cannot strictly be reduced to two dimensions
- ambulances crossing the crowd

Depending on their prevalence and impact on pedestrian flow, these effects may need to be incorporated into models to achieve fully practical applications. We categorize these distinctive features into three groups: **(i) non-standard geometry of the premises**, **(ii) diversity of goals and speed preferences**, **(iii) heterogeneity of the crowd composition**. Additionally, we identify the video recordings and specific times when these features can be observed.

File	Start	End	Description
*TopView*_1B	0:00	2:40 (end)	group of 7 standing on the right side. People are sometimes heckling or fighting, which makes the shape evolve (around 0:45).<br>Between 0:53 and 1:05, interactions between the standing group and a moving group of similar size.
*TopView*_1C	0:00	0:56	group of 3 people (2 adults and one child) standing in the middle.
*TopView*_1C	0:25	0:50	group of 3 people stopping, standing and restarting moving in the bottom right side.
*TopView*_1C	0:58	1:30	group of 2-standing people stopping, standing and resuming motion on the top side. This group is split by people passing through.
*TopView*_2A	0:00	2:41 (end)	group of standing people on the left. The group size evolves from 2 to 8 people.
*TopView*_2B	0:00	0:22	group of 2 standing people on the left
*TopView*_2B	0:45	1:12	a group of 2 walking people stops and stands on the left
*TopView*_2C	0:10	1:14	a group of 2 walking people stops and stands on the left
*TopView*_2E	0:00	0:35	2 standing groups on the top (with limited impact on flow)
*TopView*_2F	0:00	0:53	standing group of 3 people on the left border

Table 4. Situations of (temporarily) static groups observed in Dataset [3, *TopView Trajectories*].

Non-standard geometry of the premises

In contrast to the common reliance on a binary geometry, which sets a binary distinction between accessible and inaccessible spaces, the square under study exhibits regions of varying attractiveness. Notably, the vicinity of the walls where shows are projected is visibly less appealing to the crowd. Some modelling approaches have been proposed in the literature to capture this heterogeneity[13,14]. Additionally, the geometry is neither composed of straight borders nor fully two-dimensional. Knee-high bollards and waist-high steel crowd barriers restrict movement (and are thus associated with lower local density, as shown in Fig. 5) but can overlap with pedestrians in three dimensions. The coordinates of these partial obstacles are provided in Dataset [3, *Geometry*].

Diversity of goals and speed preferences

Previously, we already highlighted the complexity of the flow patterns in some sequences and the multidirectionality of the flow. Here, we focus on the effects and consequences of the diversity of intentions among spectators, notably their diverse goals and speed preferences.

Static groups of people First and foremost, numerous temporarily static groups of people can be observed, often forcing passing pedestrians to navigate around them. These groups, typically consisting of 2 to 8 individuals, were found throughout the area of interest in the *TopView* recordings. Two distinct scenarios can be identified: (i) a group moves, stops, and resumes motion; (ii) a group remains stationary for the entire footage duration. The first scenario is particularly intriguing, as it allows us to study the effects of people stopping, standing, and resuming motion over time. Table 4 summarizes our observations of such static groups, excluding those who stop for less than two seconds. Although these groups are hardly included in controlled experiments of different flow types, they disrupt the base flow, significantly impacting the dynamics. Unlike classical obstacles, these groups are more complex because they are transient, appear and disappear, and fluctuate in size and shape over time. This variability can result from the addition of new members (see Table 4, *TopView*_2A file) or specific behaviours of group members, such as heckling (see Table 4, *TopView*_1B file). Additionally, the splitting of a group of standing people by walking pedestrians has also been witnessed (see Table 4, *TopView*_1C file).

Running pedestrians Conversely, we noticed that some people were running in the instances listed in Table 5.

Marked density heterogeneities

Giant density fluctuations far exceeding the fluctuations expected in a physical system at equilibrium are common in active matter assemblies[15,16]. Here, marked density heterogeneities are conspicuous for the specific case of pedestrian assemblies. Some depleted regions (voids) are found not far from the high-density areas made of tightly packed groups in the same recording (Table 6).

File	Start	End	Nature
*TopView*_1A	2:49	2:53	2 people accelerating to reach a speed higher than the main flow from middle to right side
*TopView*_2B	0:09	0:14	3 people running from left to right

Table 5. Instances of running pedestrians observed in Dataset [3, *TopView Trajectories*].

File	Start	End	Nature
*TopView*_1A	1:09	1:18	gap, high-density in the left
*TopView*_1B	0:38	2:44	high-density in the top left (due to standing people)
*TopView*_2A	0:02	0:27	gap, high-density in the top (due to standing people)
*TopView*_2C	0:00	3:02	high-density in the top left (due to standing people)

Table 6. Voids and density heterogeneities observed in Dataset [3, *TopView Trajectories*].

Lines of people worming their way through the crowd ('serpentine' groups) In dense regions, we have often observed people worming their way through a static or counter-moving crowd and following each other, thus forming linear, snake-like structures (Table 7). People follow each other along these linear structures, dubbed serpentine groups, most probably due to the depleted channels opened in the wake of their predecessors[17] and their possible social relationships.

Similar self-organized structures have been observed in controlled experiments of people crossing static groups and dubbed 'crossing channels[12],' but overall, they have received much less attention than stable lanes in bidirectional flows or the stripes formed at the intersection of two flows. Indeed, their frequency in the empirical dataset seems to owe much to the multiple directions of pedestrians and the non-stationary character of the flow. We hypothesize that the observed transient 'snakes' could turn into stable lanes in stationary conditions and with a limited number of directions, with distinct consequences on the flow properties.

File	Start	End	Description
*TopView*_1A	0:35	0:47	lane formation on both sides
*TopView*_1A	1:11	1:28	mini-lanes: 3 people walk counter to the main flow
*TopView*_1A	1:12	2:05	serpentine group at the top
*TopView*_1B	1:05	2:40 (end)	snake/lane formation from right to left side due to a standing group; quite high density.
*TopView*_2A	0:24	0:39	a group of 6 is worming their way through a dense counter-moving crowd.
*TopView*_2B	0:27	0:40	a group of 7 is worming their way through a crowd moving in the same direction. The group splits (going from bottom to right side).
*TopView*_2B	1:12	2:00	a lane appears on the top, opposite the main flow.
*TopView*_2C	0:27	0:40	a serpentine group of 2-3 people at moderately high density (from right to left)
*TopView*_2C	0:00	0:15	a serpentine group of 9 at medium density (from right to left)

Table 7. Serpentine groups (people walking counter to the main flow and following each other in line) observed in Dataset [3, *TopView Trajectories*].

Heterogeneity of the crowd composition

Social groups Unlike the homogeneous crowds of individual agents traditionally considered by crowd modelers, the crowd at the Festival of Lights primarily consists of social groups. Some groups are quite large (see Fig. S18), even though they may be split in practice, and some are families with children. Naturally, this composition is expected to influence (at least) the microscopic dynamics at play.

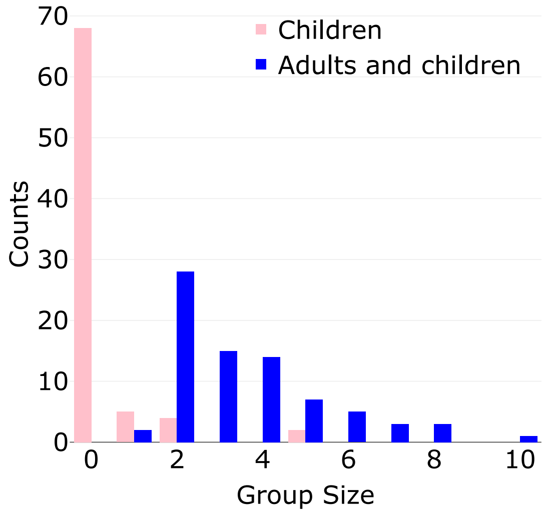

Figure S18. Histogram of social group sizes, counting both adults and children (in blue) or only the children (in light red), as reported by survey respondents around 10:30 pm to 11 pm on Friday December 9, 2022. These groups were part of the crowd preparing to enter *Place des Terreaux*. The counts refer to the number of groups. It is important to note that these stated values (survey) contain a significantly higher number of groups with at least four members compared to estimates made by the authors by means of direct street observations (Dataset [3, *Surveys*]).

Pushchairs and bikes Moreover, not all pedestrians fit the standard image of a typical pedestrian. Some navigate through crowds of varying densities while pushing strollers, while others maneuver their bicycles (Table 8). Consequently, the shape of the agent to be modelled differs widely from that of a standard pedestrian. Additionally, the density tends to be higher in front of strollers than behind them, prompting pedestrians behind the 'pushers' to frequently try to overtake them.

File	Start	End	Nature
*TopView*_1B	0:01	0:13	pedestrian with a bike
*TopView*_1B	1:33	1:48	pushchair
*TopView*_1C	3:05	3:16	pushchair
*TopView*_2A	1:25	1:53	pushchair
*TopView*_2A	2:33	2:41 (end)	pushchair
*TopView*_2B	0:49	1:12	pushchair
*TopView*_2E	0:36	0:50	pushchair
*TopView*_2F	0:59	1:06	pedestrian with a bike

Table 8. Non-standard pedestrians (pushchairs, bikes, etc.) observed in Dataset [3, *TopView Trajectories*].

Ambulances Finally, we observed instances where an ambulance needed to cross the crowd. In response, a channel opened in the crowd ahead of the vehicle to let it through[2].

References

1. Fête des Lumière 2022 Official Map. weblink.
2. Largeview ambulance, URL https://www.youtube.com/watch?v=1zqpJRnAqsM.
3. Dufour, O. *et al.* Dense Crowd Dynamics and Pedestrian Trajectories: A Multiscale Field Study at the Fête des Lumières in Lyon, https://doi.org/10.5281/zenodo.13830435 (2024).
4. Chraibi, M. & Dufour, O. MADRAS-data-app, https://doi.org/10.5281/zenodo.10694867, URL https://go.fzj.de/madras-app (2024).
5. Schrödter, Tobias & The PedPy Development Team. Pedestriandynamics/pedpy: v1.0.2, https://doi.org/10.5281/zenodo.10016938 (2023).
6. Nicolas, A. & Hassan, F. H. Social groups in pedestrian crowds: review of their influence on the dynamics and their modelling. *Transp. A: transport science* **19**, 1970651, https://doi.org/10.1080/23249935.2021.1970651 (2023).
7. Vanumu, L. D., Ramachandra Rao, K. & Tiwari, G. Fundamental diagrams of pedestrian flow characteristics: A review. *Eur. transport research review* **9**, 1–13, https://doi.org/10.1007/s12544-017-0264-6 (2017).
8. Wirz, M. *et al.* Probing crowd density through smartphones in city-scale mass gatherings. *EPJ Data Sci.* **2**, 1–24, https://doi.org/10.1140/epjds17 (2013).
9. Jiang, R. *et al.* Traffic experiment reveals the nature of car-following. *PloS one* **9**, e94351, https://doi.org/10.1371/journal.pone.0094351 (2014).
10. Van Den Berg, J., Guy, S. J., Lin, M. & Manocha, D. Reciprocal n-body collision avoidance. In *Robotics Research: The 14th International Symposium ISRR*, 3–19, https://doi.org/10.1007/978-3-642-19457-3_1 (Springer, 2011).
11. Karamouzas, I., Sohre, N., Narain, R. & Guy, S. J. Implicit crowds: Optimization integrator for robust crowd simulation. *ACM Transactions on Graph. (TOG)* **36**, 1–13, https://doi.org/10.1145/3072959.3073705 (2017).
12. Wang, J., Lv, W., Jiang, H., Fang, Z. & Ma, J. Exploring crowd persistent dynamism from pedestrian crossing perspective: An empirical study. *Transp. research part C: emerging technologies* **157**, 104400, https://doi.org/10.1016/j.trc.2023.104400 (2023).
13. Helbing, D., Keltsch, J. & Molnar, P. Modelling the evolution of human trail systems. *Nature* **388**, 47–50, https://doi.org/10.1038/40353 (1997).
14. Echeverría-Huarte, I. & Nicolas, A. Body and mind: Decoding the dynamics of pedestrians and the effect of smartphone distraction by coupling mechanical and decisional processes. *Transp. research part C: emerging technologies* **157**, 104365, https://doi.org/10.1016/j.trc.2023.104365 (2023).
15. Dey, S., Das, D. & Rajesh, R. Spatial structures and giant number fluctuations in models of active matter. *Phys. review letters* **108**, 238001, https://doi.org/10.1103/PhysRevLett.108.238001 (2012).
16. Manning, M. L. Essay: Collections of deformable particles present exciting challenges for soft matter and biological physics. *Phys. Rev. Lett.* **130**, 130002, https://doi.org/10.1103/PhysRevLett.130.130002 (2023).
17. Nicolas, A., Kuperman, M., Ibañez, S., Bouzat, S. & Appert-Rolland, C. Mechanical response of dense pedestrian crowds to the crossing of intruders. *Sci. reports* **9**, 105, https://doi.org/10.1038/s41598-018-36711-7 (2019).
